# Triglyceride-glucose index predicts postoperative overall survival in hepatocellular carcinoma: a retrospective cohort study

**DOI:** 10.1007/s12672-024-01541-9

**Published:** 2024-11-13

**Authors:** Gao-Min Liu, Wen-Biao Zhu, Ji-Wei Xu

**Affiliations:** 1 Meizhou Clinical Institute of Shantou University Medical College, No. 38 Huangtang Road, Meizhou, 514000 China; 2 Department of Hepatobiliary Surgery, Department of Pathology, Meizhou People’s Hospital, No. 38 Huangtang Road, Meizhou, 514000 China

**Keywords:** Hepatocellular carcinoma, Hepatectomy, TyG, Survival, Nomogram

## Abstract

**Background:**

Insulin resistance is important in hepatocellular carcinoma (HCC) carcinogenesis and progression. The triglyceride-glucose (TyG) index, triglyceride to high-density lipoprotein cholesterol (TG/HDL-c) ratio or TyG-body mass index (TyG-BMI) are three non-invasive parameters for insulin resistance. However, their prognostic role in HCC patients undergoing hepatectomy remains unclear.

**Materials and methods:**

HCC patients who underwent hepatectomy at the Meizhou People’s Hospital from May 2011 to February 2023 were retrospectively explored. Patients were classified into high and low groups based on different TyG, TG/HDL-c, and TyG-BMI indices. The prognostic role of TyG, TG/HDL-c, and TyG-BMI was evaluated using Kaplan–Meier analysis and Cox regression models. A nomogram incorporating significant prognostic factors was constructed and validated.

**Results:**

A lower TyG, lower TG/HDL-c, and lower TyG-BMI were linked to worse overall survival (OS) in HCC patients. Multivariate analysis indicated the TyG index, but not the TG/HDL-c and TyG-BMI index, could independently predict HCC OS. The nomogram incorporating the TNM stage and TyG index demonstrated good calibration, discriminative ability, and clinical benefit for predicting OS in HCC patients.

**Conclusions:**

The TyG index could independently predict HCC OS after hepatectomy in this cohort. The nomogram incorporating the TyG index may aid in the prognosis management of HCC.

**Supplementary Information:**

The online version contains supplementary material available at 10.1007/s12672-024-01541-9.

## Introduction

Hepatocellular carcinoma (HCC) is typically developed from chronic viral hepatitis and liver cirrhosis in Asia [1]. The advancements in earlier diagnosis, targeted therapies, and immunotherapies have dramatically improved the overall prognosis for HCC. Currently, hepatectomy remains one of the most effective curative treatments for HCC. However, post-operative recurrence and metastasis are major challenges that liver cancer patients have to face after hepatectomy [2]. Identifying potential risk factors and implementing early interventions is critical to further improving the prognosis of patients after HCC resection.

Under certain pathological conditions, the body's ability to utilize insulin to promote glucose metabolism is impaired, leading the body to produce excessive insulin to maintain stable blood glucose levels, a condition known as insulin resistance (IR). IR participates in the development and progression of many metabolic-related disorders, such as diabetes, nonalcoholic fatty liver disease (NAFLD), and various types of cancer, including HCC [3, 4]. The standard hyperglycemic clamp method is cumbersome and inconvenient when evaluating IR. Several more straightforward non-invasive parameters, such as HOMA2-IR, triglyceride-glucose index (TyG), triglyceride/high-density lipoprotein cholesterol ratio (TG/HDL-c), and triglyceride-glucose index–body mass index (TyG-BMI) index, have been developed for clinical assessment of IR [5]. These novel insulin resistance indices provide more convenient alternatives to the hyperglycemic clamp. The role of these non-invasive parameters in tumor prognosis remains controversial. For example, the TyG index was found to be an independent risk factor for overall survival (OS) in pancreatic cancer and renal cell carcinoma; however, in gastric cancer, it was a protective factor for OS [6–8]. The TyG index has been independently associated with a high risk of incident metabolic-associated fatty liver disease [9]. In HCC, a high TyG index promoted the development of HCC [10]. However, as far as we know, no study has examined the long-term prognostic role of TyG, TG/HDL-c, and TyG-BMI levels in HCC post hepatectomy.

Therefore, we retrospectively explored whether TyG, TG/HDL-c, and TyG-BMI index were independent prognostic factors for HCC survival post hepatectomy.

## Materials and methods

### Study population

We retrospectively retrieved 415 patients with HCC who underwent hepatectomy as initial treatment at the Meizhou People’s Hospital from May 2011 to February 2023 for analysis. This study fulfilled the Declaration of Helsinki. The Ethics Committee of Meizhou People's Hospital approved this study and waived the requirement for written informed consent (2023-C-95).

### Inclusion and exclusion criteria

The inclusion criteria were: (I) received hepatectomy as the initial treatment; (II) pathologically diagnosed with HCC; (III) biochemical blood examination performed within one week before hepatectomy; (IV) clinicopathological data, especially data concerning TyG and survival, were available.

**Fig. 1:**
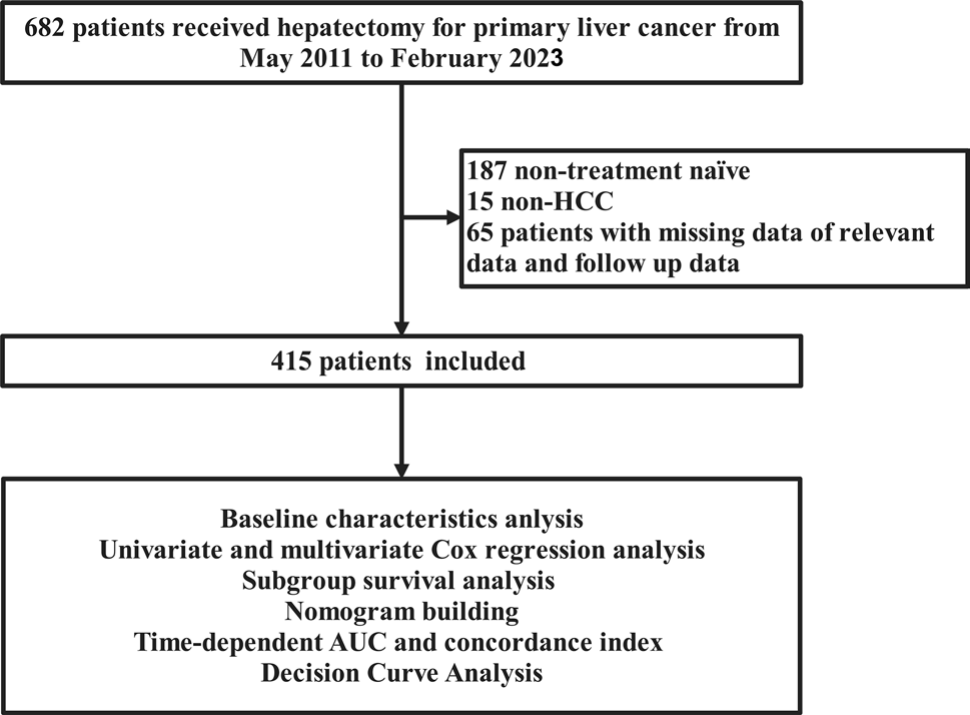
The flow chart of the study

The exclusion criteria were: (I) history of multiple primary cancers; (II) received other antitumor treatments, including chemotherapy, radiotherapy, or intervention therapy before hepatectomy. The patient selection process was depicted in a flowchart, as shown in Fig. 1.

### Data collection

The retrieved data included: (I) Demographic characteristics: age, sex, weight, height, background liver disease (hepatitis, cirrhosis, portal hypertension Child–Pugh score); (II) preoperative blood parameters: albumin (ALB), alanine aminotransferase (ALT), alpha-fetoprotein (AFP), fasting plasma glucose (FPG), glutamyl transpeptidase (GGT), high-density lipoprotein cholesterol (HDL-c), total bilirubin (TBIL), total cholesterol (TC), triglycerides (TG), hepatitis B surface antigen (HBsAg); (III) surgical conditions: intraoperative bleeding, surgical approach, major resection, and anatomic resection; (IV) tumor characteristics: number, grade, capsule, vascular invasion, microvascular invasion (MVI), and TNM stage; (V) OS from outpatient or telephone follow-up. Patients were staged with the International Union Against Cancer TNM classification system (version 8th). Since the study spans more than ten years, to ensure consistency in pathological diagnoses, all case slides were reviewed by two independent pathologists based on the latest guideline standards. A major hepatectomy was characterized as the removal of four or more adjacent segments of the liver [11]. All surgical procedures and postoperative follow-up schedules adhered to the guidelines of the various editions of China’s Primary Liver Cancer Diagnosis and Treatment Guidelines [12]. All patients underwent regular outpatient and telephone follow-up. The last follow-up time is July 7th, 2023. OS was defined as the time from initial hepatectomy to death or the last follow-up.

### Calculation of TyG, TG/HDL-c, and TyG-BMI

The formula for TYG was ln [TG (mg/dL) × FPG (mg/dL)/2]. The formula for TG/HDL-c index was TG (mg∕dL) / HDL -c (mg∕dL); The formula for TyG -BMI index was TyG × Weight (kg)∕Height^2^ (m^2^) (kg∕m2) as previous study [6]. The optimal cut-off point of these indexes was calculated using the R package “survminer” version 0.4.9 and then stratified patients into high and low groups.

### Statistical analysis

All analyses were conducted using R software, version 4.3.1 (http://www.r-project.org). Qualitative variables are expressed as mean ± standard deviation or median (interquartile spacing), and quantitative variables are expressed as counts (percentages). Comparisons of qualitative variables were performed using the Mann–Whitney U test or independent samples t-test as appropriate. Qualitative variables, on the other hand, were assessed using the Pearson χ2 test or Fisher's exact test, as applicable. Survival difference was compared with the Kaplan–Meier curve and the log-rank test using the R software package “survival” version 3.5–5. Univariate and subsequent forward stepwise multivariate Cox proportional hazards regression analyses were performed to identify risk factors. The interaction P value was assessed by conducting a likelihood ratio test that compared the main regression analysis with the interaction model, utilizing the R package “jstable” version 1.6.5. A two-sided P < 0.05 was considered statistically significant.

### Building and internal validation of the prognostic nomogram

A nomogram [13] containing all independent prognostic factors identified by multivariate Cox proportional hazards regression analyses was created using the R package "regplot" version 1.1. Each predictor had its own score, and the overall score reflected the cumulative sum of the scores from the predictors mentioned above. The calibration power of nomograms was assessed by plotting calibration maps using a bootstrap technique with 1000 replicate samples. The discriminatory power of the nomograms was evaluated using receiver operating characteristic (ROC) analysis over time and the C index. The clinical benefit was evaluated using decision curve analysis (DCA) [14].

## Results

### Baseline clinicopathological characteristics

This retrospective study included 415 HCC patients who had received hepatectomy as an initial treatment in our hospital. Of the 415 patients enrolled, 372 (89.6) were male and 43 (10.4) were female; 346 (83.4) were HBsAg positive, 290 (69.9) had cirrhosis, and 65 (15.7) had preoperative diabetes mellitus. 313 (75.4) had stage I + II, and 102 (24.6) had stage III + IV. The median [IQR] follow-up was 30.34 [20.87–51.76] months. A total of 20.48% of the patient data were censored, meaning these patients were still alive at the time of the last follow-up and had not experienced the events defined by the study. Among them, 17.89% of the censored data occurred in stage I + II, while 28.43% occurred in stage III + IV. A total of 51 patients (13.7%) died, with 26 (8.31%) of these cases occurring in stage I + II and 31 (30.39%) in stage III + IV. The median [IQR] TyG, TG/HDL-c, and TyG-BMI indices were 7.05 [6.80, 7.44], 0.95 [0.69, 1.43], and 164.79 [150.34, 185.28], respectively. Table 1 presents the detailed clinicopathological characteristics of the high and low-TyG groups. BMI, hypertension, preoperative diabetes mellitus, HBsAg positivity, AFP, ALB, GGT, FPG, TG, HDL-C, tumor size, number of tumors, TG/HDL-c, and TyG-BMI differed significantly between the high and low TyG groups. There were no significant differences in other clinicopathologic features, especially TNM staging.

**Table 1:** Baseline characteristics of included patients

Characteristics	Overall	TyG^high^	TyG^low^	P
	415	263	152	
Age (mean (SD)) (Years)	58.00 (11.07)	60.00 [52.00, 66.00]	57.50 [48.00, 65.00]	0.071
Gender (%)				
Female	43 (10.4)	27 ( 10.3)	16 ( 10.5)	1.000
Male	372 (89.6)	236 ( 89.7)	136 ( 89.5)	
BMI (median [IQR]) (Kg/m2)	22.80 (3.42)	23.34 [21.27, 24.98]	21.28 [19.44, 23.54]	** < 0.001**
Hypertension (%)				
No	339 (81.7)	204 ( 77.6)	135 ( 88.8)	**0.006**
Yes	76 (18.3)	59 ( 22.4)	17 ( 11.2)	
Diabetes (%)				
No	350 (84.3)	203 ( 77.2)	147 ( 96.7)	** < 0.001**
Yes	65 (15.7)	60 ( 22.8)	5 ( 3.3)	
HBsAg (%)				
Negative	69 (16.6)	53 ( 20.2)	16 ( 10.5)	**0.016**
Positive	346 (83.4)	210 ( 79.8)	136 ( 89.5)	
Cirrhosis (%)				
No	125 (30.1)	78 ( 29.7)	47 ( 30.9)	0.874
Yes	290 (69.9)	185 ( 70.3)	105 ( 69.1)	
Portal hypertension (%)				
No	326 (78.6)	207 ( 78.7)	119 ( 78.3)	1.000
Yes	89 (21.4)	56 ( 21.3)	33 ( 21.7)	
Child–Pugh score (%)				
5	287 (69.2)	190 ( 72.2)	97 ( 63.8)	0.093
6	128 (30.8)	73 ( 27.8)	55 ( 36.2)	
AFP (median [IQR]) (ng/ml)	2243.80 (5346.48)	19.18 [4.29, 461.56]	131.12 [6.16, 1880.45]	**0.006**
ALB (mean (SD)) (g/L)	40.88 (5.15)	41.90 [38.20, 45.30]	40.30 [37.20, 43.25]	**0.006**
Tbil (mean (SD)) (umol/L)	18.76 (22.67)	14.90 [11.00, 22.05]	15.10 [11.57, 20.62]	0.923
GGT (mean (SD)) (U/L)	116.08 (343.27)	61.00 [33.00, 120.50]	48.00 [28.00, 100.50]	**0.020**
ALT (mean (SD)) (U/L)	55.31 (81.53)	37.00 [26.00, 55.00]	36.00 [25.00, 53.00]	0.429
FPG (mean (SD)) (mmol/L)	5.85 (2.33)	5.63 [5.04, 6.90]	4.54 [4.20, 5.14]	** < 0.001**
TG (mean (SD)) (mmol/L)	1.21 (0.86)	1.22 [1.01, 1.67]	0.72 [0.61, 0.86]	** < 0.001**
HDL-C (mean (SD)) (mmol/L)	1.46 (0.71)	1.25 [1.02, 1.53]	1.39 [1.17, 1.73]	**0.001**
TG/HDL-c (mean (SD))	1.01 (1.28)	0.95 [0.69, 1.43]	0.54 [0.39, 0.65]	** < 0.001**
TyG-BMI (mean (SD))	156.65 (30.42)	164.79 [150.34, 185.28]	133.32 [120.34, 148.83]	** < 0.001**
Tumor size (mean (SD)) (cm)	5.63 (3.26)	4.70 [3.00, 6.55]	5.90 [3.58, 9.00]	** < 0.001**
Tumor size (cm) (%)				
< 5	195 (47.0)	136 ( 51.7)	59 ( 38.8)	**0.015**
≥ 5	220 (53.0)	127 ( 48.3)	93 ( 61.2)	
Tumor number (%)				
Single	328 (79.0)	217 ( 82.5)	111 ( 73.0)	**0.031**
Multiple	87 (21.0)	46 ( 17.5)	41 ( 27.0)	
Tumor Capsule (%)				
Complete	367 (88.4)	230 ( 87.5)	137 ( 90.1)	0.507
Incomplete	48 (11.6)	33 ( 12.5)	15 ( 9.9)	
Vascular invasion (%)				
No	310 (74.7)	204 ( 77.6)	106 ( 69.7)	0.099
Yes	105 (25.3)	59 ( 22.4)	46 ( 30.3)	
MVI (%)				
M0	262 (63.1)	167 ( 63.5)	95 ( 62.5)	0.967
M1	90 (21.7)	56 ( 21.3)	34 ( 22.4)	
M2	63 (15.2)	40 ( 15.2)	23 ( 15.1)	
Tumor Grade (%)				
1	19 ( 4.9)	12 ( 4.9)	7 ( 4.8)	0.670
2	264 (67.5)	169 ( 69.3)	95 ( 64.6)	
3	104 (26.6)	60 ( 24.6)	44 ( 29.9)	
4	4 ( 1.0)	3 ( 1.2)	1 ( 0.7)	
Anatomical resection (%)				
No	178 (42.9)	122 ( 46.4)	56 ( 36.8)	0.073
Yes	237 (57.1)	141 ( 53.6)	96 ( 63.2)	
Surgical approach (%)				
Conversion	45 (10.8)	23 ( 8.7)	22 ( 14.5)	0.122
Laparoscopic	251 (60.5)	167 ( 63.5)	84 ( 55.3)	
Open	119 (28.7)	73 ( 27.8)	46 ( 30.3)	
Major resection (%)				
No	286 (68.9)	189 ( 71.9)	97 ( 63.8)	0.110
Yes	129 (31.1)	74 ( 28.1)	55 ( 36.2)	
Intraoperative_bleeding (mean (SD)) (ml)	340.14 (507.76)	200.00 [90.00, 400.00]	200.00 [100.00, 400.00]	0.642
TNM stage (%)				
I + II	313 (75.4)	205 ( 77.9)	108 ( 71.1)	0.146
III + IV	102 (24.6)	58 ( 22.1)	44 ( 28.9)	

*ALB* albumin, *ALT* alanine aminotransferase, *AFP* alpha-fetoprotein, *BMI* body mass index, *FPG* fasting plasma glucose, *HDL-c* high-density lipoprotein cholesterol, *TBIL* total bilirubin, *TC* total cholesterol, *TG* triglycerides, *TG/HDL-c* triglycerides / high-density lipoprotein cholesterol ratio, *TyG* triglyceride-glucose index, *TyG-BMI* triglyceride-glucose index–body mass index, *GGT* glutamyl transpeptidase, *HBsAg* hepatitis B surface antigen, *MVI* microvascular invasion, *TNM* tumor node metastasis classification

Bold P: P < 0.05

### Prognostic value of the TyG, TG/HDL-c, and TyG-BMI

**Fig. 2:**
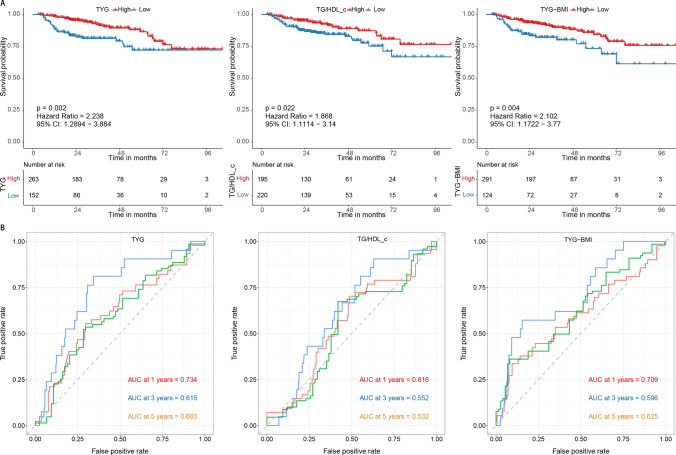
The Kaplan–Meier and receiver operator characteristic curves for different IR indexes. **A** The Kaplan-Meier curve for overall survival by different IR indexes; **B** The receiver operator characteristic curves for different IR indexes. *IR* insulin resistance; TG/HDL-c: triglycerides / high-density lipoprotein cholesterol ratio, *TyG* triglyceride-glucose index, *TyG-BMI* triglyceride-glucose index-body mass index.

We first assessed whether the TyG, TG/HDL-c, and TyG-BMI indexes could differentiate patients’ prognoses. The cut-off points of these three parameters were set as 6.58, 0.78, and 138.34. The results showed that low TyG (HR = 2.238, 95% CI 1.289–3.884, p = 0.002), low TG/HDL-c (HR = 1.868, 95% CI 1.111–3.140, p = 0.022), and low TyG-BMI (HR = 2.102, 95% CI 1.172–3.770, p = 0.004) were significantly linked to worse OS (Fig. 2A). The 1-, 3-, and 5 year AUC of the TyG were 0.734, 0.615, and 0.603, respectively. The 1-, 3-, and 5 year AUC of the TG/HDL-c were 0.616, 0.552, and 0.532, respectively. The 1-, 3-, and 5 year AUC of the TyG-BMI were 0.709, 0.596, and 0.625, respectively (Fig. 2B). All three parameters had modest prognostic efficacy, especially in 1 year OS of HCC patients.

### TyG, but not TG/HDL-c and TyG-BMI, was an independent factor of OS.

**Fig. 3:**
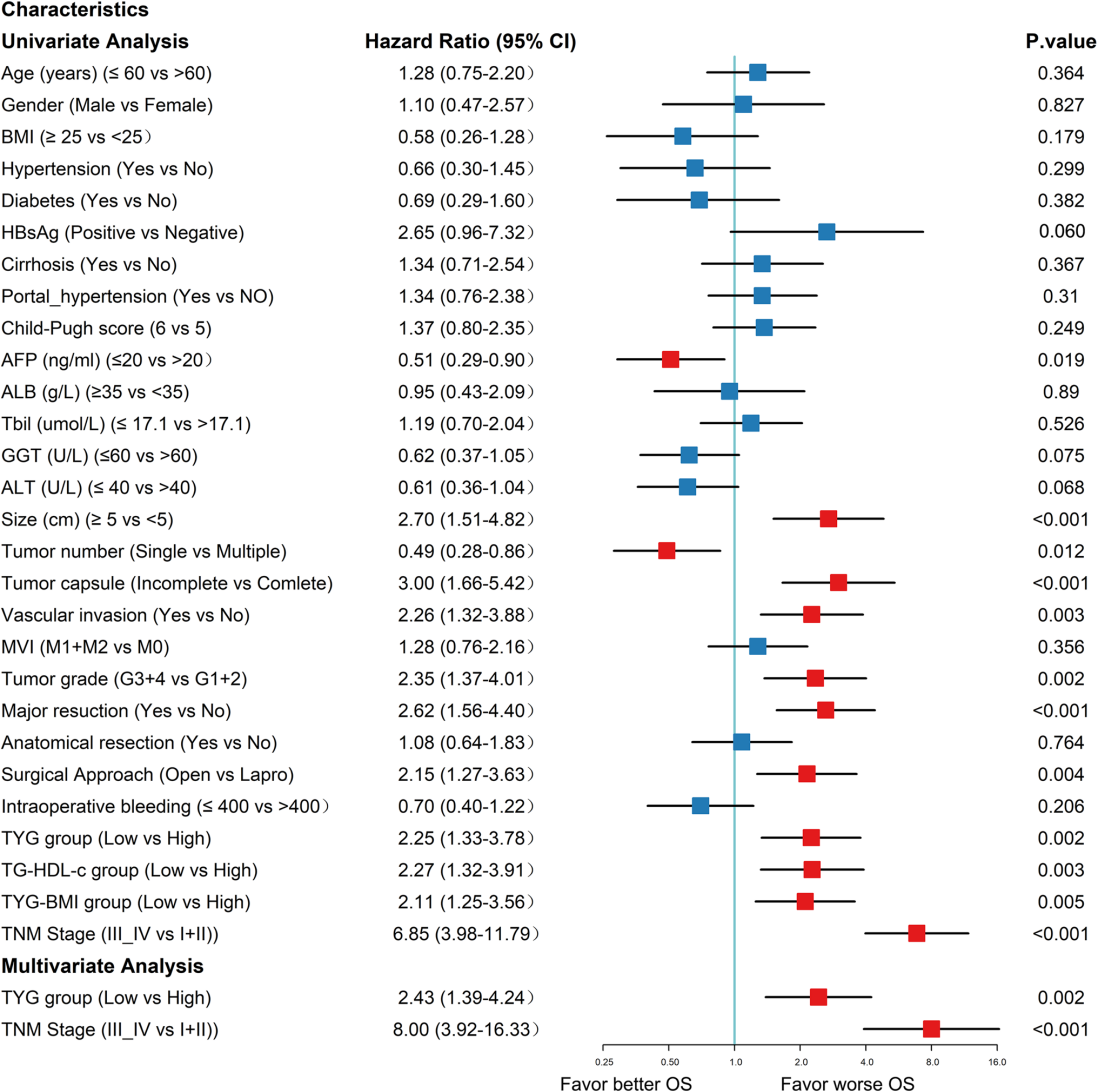
Forrest plot of the univariate and multivariate Cox regression analysis in HCC. *ALB* albumin, *ALT* alanine aminotransferase, *AFP* alpha-fetoprotein, *BMI* body mass index, *FPG* fasting plasma glucose, *HDL-c* high-density lipoprotein cholesterol, *TBIL* total bilirubin, *TC* total cholesterol, *TG* triglycerides, *TG/HDL-c* triglycerides / high-density lipoprotein cholesterol ratio, *TyG* triglyceride-glucose index, *TyG-BMI* triglyceride-glucose index–body mass index, *GGT* glutamyl transpeptidase, *HBsAg* hepatitis B surface antigen, *MVI* microvascular invasion, *TNM* tumor node metastasis classification

**Fig. 4:**
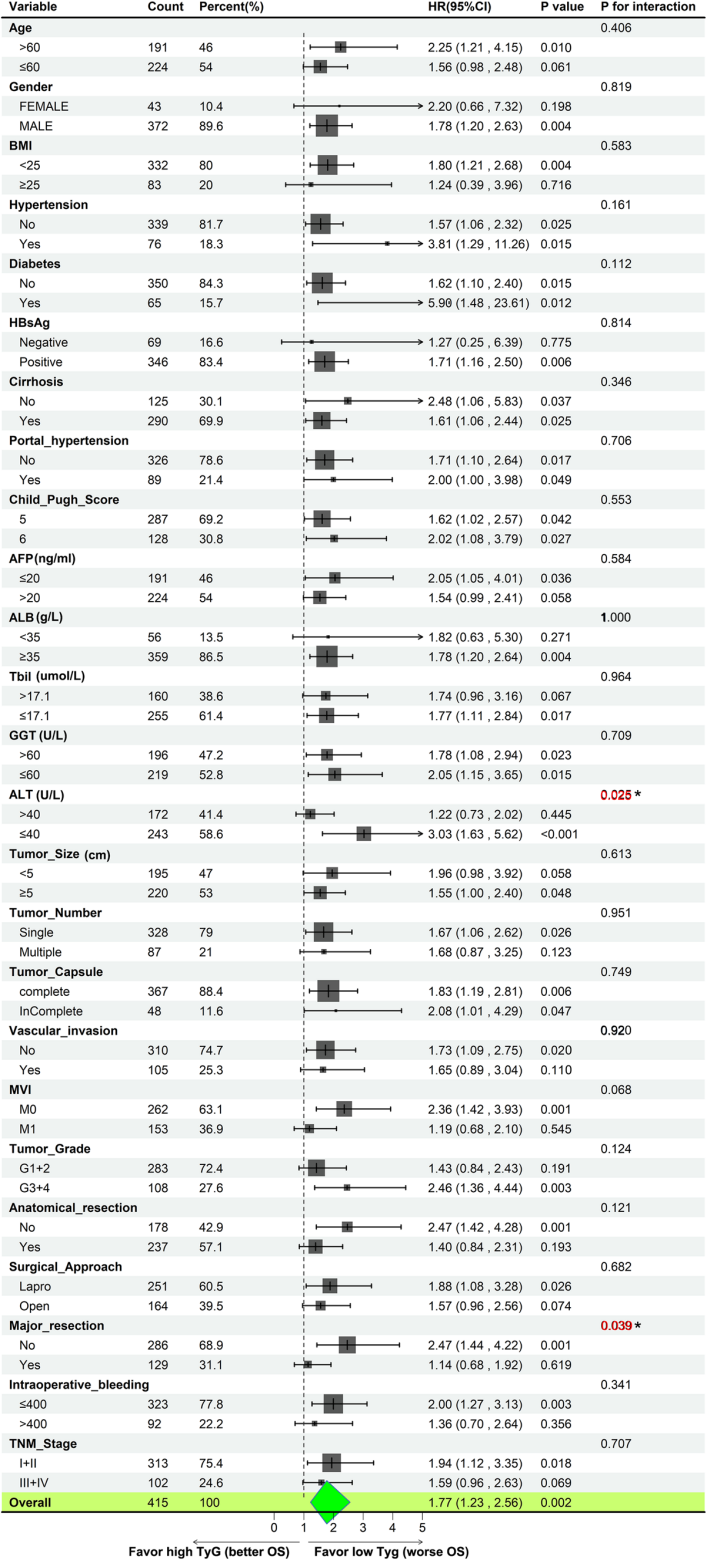
Forrest plot of the subgroup survival using univariate Cox regression analysis in HCC The horizontal axis represented the HR, with larger values indicating higher risk; the vertical axis displayed the results for each subgroup. The P for interaction assessed the consistency of the proportional risk effect of the TyG index on mortality across different subgroups using likelihood ratio tests. *ALB* albumin, *ALT* alanine aminotransferase, *AFP* alpha-fetoprotein, *BMI* body mass index, *FPG* fasting plasma glucose, *HDL-c* high-density lipoprotein cholesterol, *TBIL* total bilirubin, *TC* total cholesterol, *TG* triglycerides, *TG/HDL-c* triglycerides / high-density lipoprotein cholesterol ratio, *TyG* triglyceride-glucose index, *TyG-BMI* triglyceride-glucose index-body mass index, *GGT* glutamyl transpeptidase, *HBsAg* hepatitis B surface antigen, *MVI* microvascular invasion, *TNM* tumor node metastasis classification.

Univariate analysis showed that preoperative AFP (HR = 0.51; 95% CI 0.29–0.90; p = 0.019), tumor size (HR = 2.70; 95% CI 1.51–4.82; p < 0.001), tumor number (HR = 0.49, 95% CI 0.28–0.86; p = 0.012), tumor capsule (HR = 3.00; 95% CI 1.66–5.42; p < 0.001), vascular invasion (HR = 2.26; 95% CI 1.32–3.88; p = 0.003), tumor grade (HR = 2.35; 95% CI 1.37–4.01; p = 0.002), major resection (HR = 2.62; 95% CI 1.56–4.40; p < 0.001), surgical approach (HR = 2.15; 95% CI 1.27–3.63; p = 0.004), low TyG index (HR = 2.25; 95% CI 1.33–3.78; p = 0.002), low TG/HDL-c (HR = 1.88; 95% CI 1.09–3.24; p = 0.024), low TyG-BMI (HR = 2.11; 95% CI 1.25–3.56; p = 0.005), and TNM stage (HR = 6.85; 95% CI 3.98–11.79; p < 0.001) significantly predicted OS. Furthermore, the multivariate analysis suggested that, in addition to the TNM stage (HR = 8.00; 95% CI 3.92–16.33; p < 0.001), low TyG index (HR = 2.43, 95% CI 1.39–4.24, p = 0.002) but not TG/HDL-c (HR = 0.90; 95% CI 0.41–1.96; p = 0.791) and TyG-BMI (HR = 1.51; 95% CI 0.81–2.82; p = 0.191), were independent prognostic factors for OS (Fig. 3). In exploratory analyses, Fig. 4 showed the impact of a lower TyG index on survival prognosis across different subgroups using Cox proportional hazards regression analysis. Specifically, the horizontal axis represented the HR, with larger values indicating higher risk; the vertical axis displayed the results for each subgroup. The P for interaction assessed the consistency of the proportional risk effect of the TyG index on mortality across different subgroups. The proportional effect of lower TyG index on mortality was consistent across 23 pre-specified subgroups but not in the ALT (P for interaction: 0.025) and major resection (P for interaction: 0.039) subgroups.

### Cox regression analyses, nomogram building, and DCA curve for OS

**Fig. 5:**
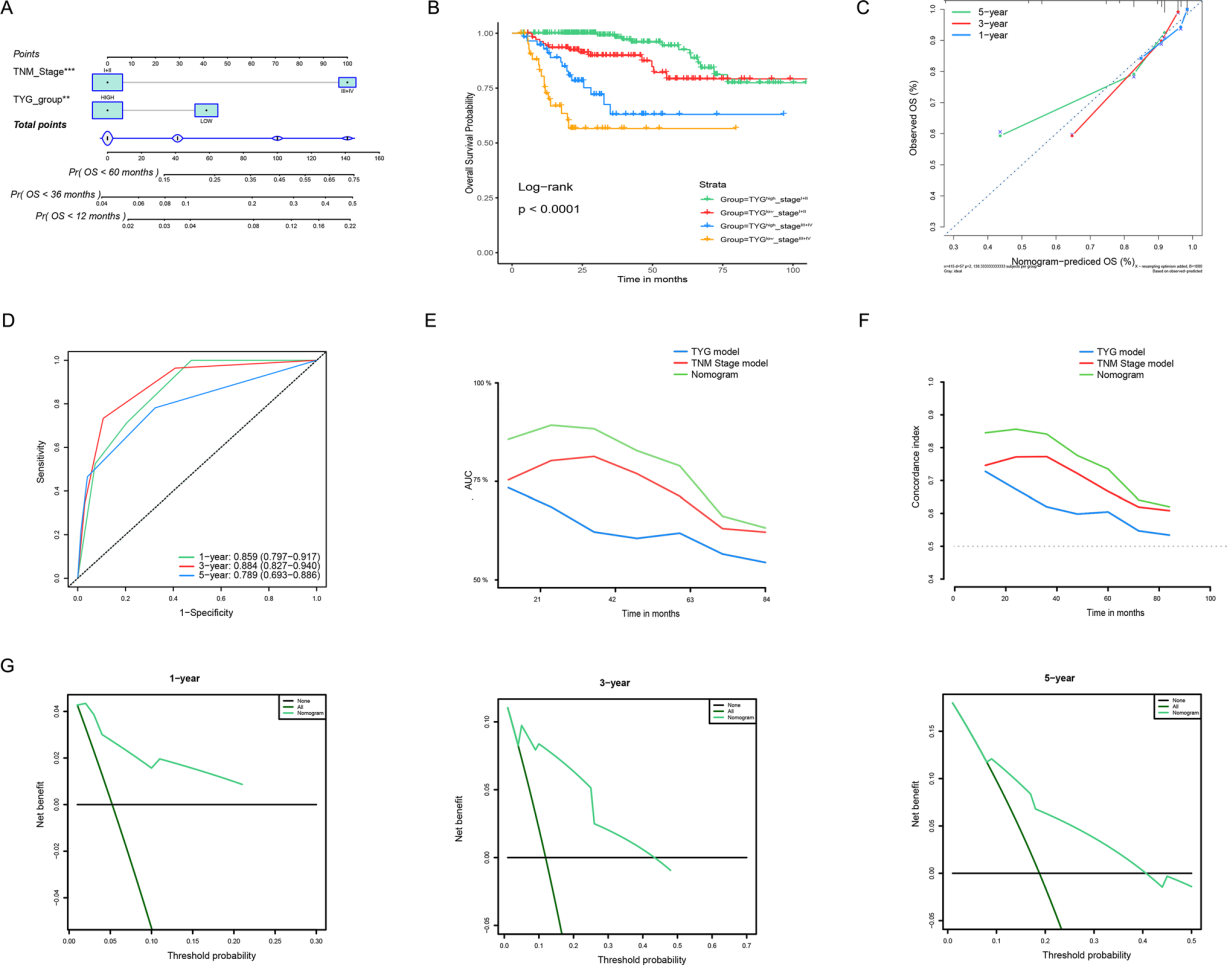
Building the nomogram predicting overall survival for HCC patients **A **The nomogram plot was built based on three independent prognostic factors in HCC. **B** The Kaplan-Meier curve showed the survival difference between high and low-risk patients stratified by the nomogram. **C** The calibration plot for internal validation of the nomogram. **D** The time-dependent AUC curves of the nomogram. **E** The time-dependent AUC curves compare the nomogram, TNM, and TyG models. **F** The time-dependent concordance index curves compare the nomogram, TNM, and TyG models. **G** The DCA curves of the nomograms.

A nomogram containing the TNM stage and TyG index was developed (Fig. 5A). The nomogram clearly contains four key nodes, which, upon careful observation, represent from left to right: TyG^low^stage^III+IV^, TyG^high^stage^III+IV^, TgG^low^stage^I+II^, TgG^high^stage^I+II^. Patients in different nomogram subgroup had significantly different OS, with TyG^low^stage^III+IV^ showing the poorest prognosis, while TgG^high^stage^I+II^ had the best prognosis (P < 0.001) (Fig. 5B). Calibration plots suggest that column plots are better predictors of short-term prognosis, especially 1 year OS (Fig. 5C). The 1-, 3-, and 5-year AUC of the nomogram were 0.859 (95% CI 0.797–0.917), 0.884 (95% CI 0.827–0.940), and 0.789 (95% CI 0.693–0.886), respectively (Fig. 5D). The C-index was 0.612 (95% CI 0.575–0.709), 0.745 (95% CI 0.684–0.807) and 0.816 (95% CI 0.773–0.860) for the TyG model, TNM stage mode, and the nomogram model, respectively. The nomogram was further suggested to be better than the TNM and TyG index models alone by AUC and C-index curves. (Figs. 5E, F). DCA curve indicated that the nomogram provided clear clinical benefits in predicting HCC OS (Fig. 5G).

### TNM stage I + II subgroup analysis

**Fig. 6:**
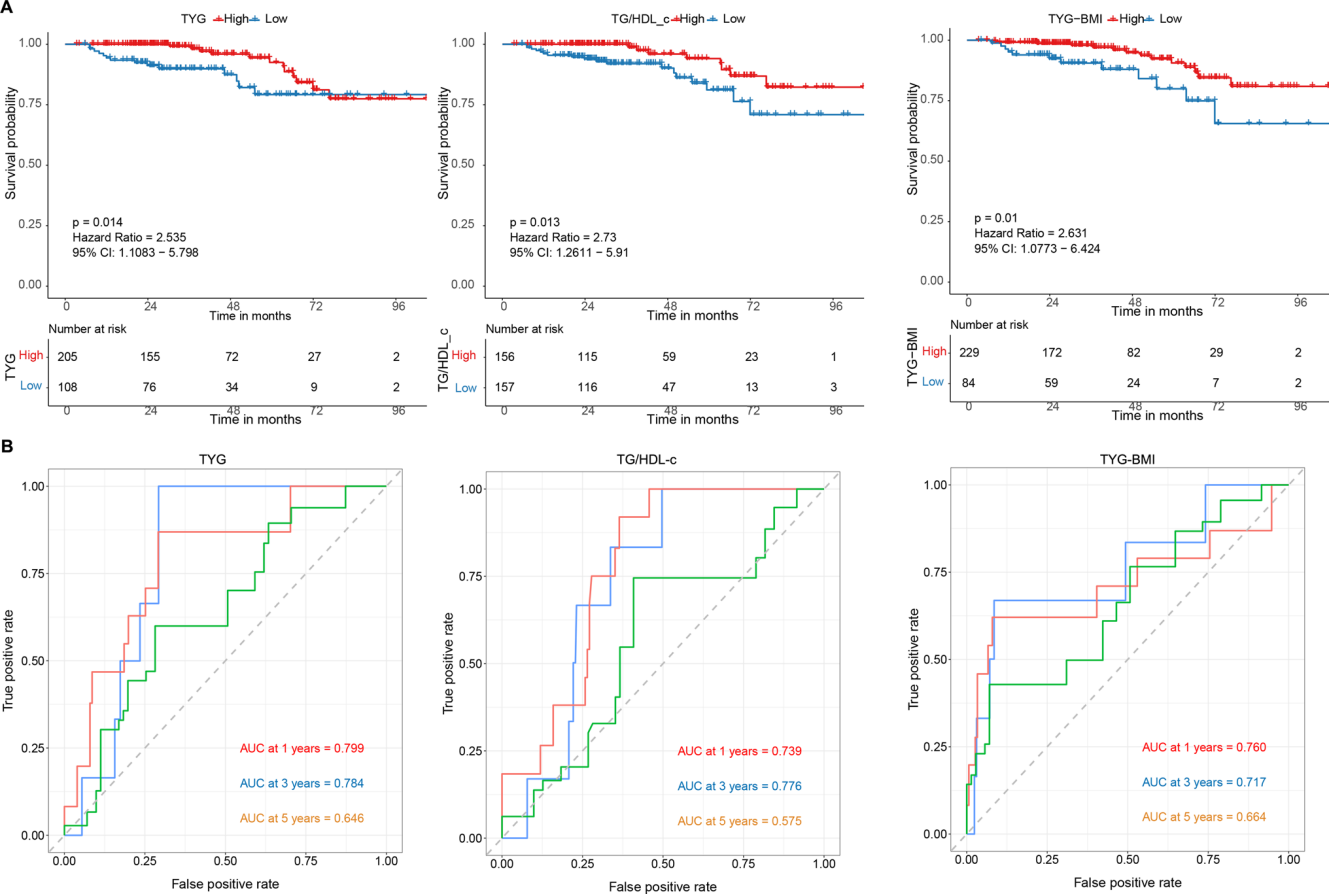
The Kaplan–Meier and receiver operator characteristic curves for different IR indexes in stage I + II patients. **A** The Kaplan-Meier curve for overall survival by different IR indexes; **B** The receiver operator characteristic curves for different IR indexes. *IR* insulin resistance, TG/HDL-c: triglycerides / high-density lipoprotein cholesterol ratio, *TyG* triglyceride-glucose index, *TyG-BMI* triglyceride-glucose index-body mass index.

We then explored the clinical significance of the TyG, TG/HDL-c, and TyG-BMI in TNM stage I + II patients merely, which usually received hepatectomy as the standard treatment. Similar to that observed in the overall population BMI, hypertension, preoperative diabetes mellitus, HBsAg positivity, GGT, FPG, TG, HDL-C, number of tumors, TG/HDL-c, and TyG-BMI differed significantly between the high and low TyG groups (Supplementary Table 1). The Kaplan Meier analyses indicated that low TyG (HR = 2.532 95% CI 1.108–5.798, p = 0.014), low TG/HDL-c (HR = 2.73, 95% CI 1.261–5.910, p = 0.013), and low TyG-BMI (HR = 2.631, 95% CI 1.077–6.424, p = 0.010) were significantly linked to worse OS (Fig. 6A). The 1-, 3-, and 5-year AUC of the TyG were 0.799, 0.784, and 0.646, respectively. The 1-, 3-, and 5-year AUC of the TG/HDL-c were 0.739, 0.776, and 0.575, respectively. The 1-, 3-, and 5-year AUC of the TyG-BMI were 0.760, 0.717, and 0.664, respectively (Fig. 6B). In the stage I + II subgroup, the prognostic predictive performance of the three parameters was improved compared to the overall population. The univariate analysis revealed that a low TyG index (HR = 2.55; 95% CI 1.18–5.53; p = 0.018), low TG/HDL-c (HR = 2.76; 95% CI 1.20–6.38; p = 0.017), and low TyG-BMI (HR = 2.64; 95% CI 1.22–5.71; p = 0.014) were significant predictors of overall survival (OS). However, the multivariate analysis did not identify any independent prognostic factors (Supplementary Fig. 1).

## Discussion

In recent years, advances in multiple disciplinary treatment strategies and targeted/immunologic agents have greatly improved the prognosis of patients and brought the treatment of hepatocellular carcinoma to a new level. Nevertheless, postoperative recurrence and metastasis remain complex problems that need to be handled [15]. Many prognostic factors, including clinical or genetic factors, have been identified to help HCC OS prediction [16–18]. IR has been well-known to facilitate HCC development. For example, the TyG index, a non-invasive predictor of IR, was suggested to promote HCC carcinogenesis in those with HBV-related cirrhosis recently [10]. However, the prognostic role of these predictors in hepatocellular carcinoma remains unclear. In this study, we found that TyG was significantly correlated with several well-known prognostic factors, such as AFP, tumor size, and number, but not with the TNM stage. In addition to the TNM stage, the TyG index independently predicted postoperative OS for HCC. Subsequent internal validation further demonstrated the good calibration, discriminative ability, and clinical benefit of the nomogram combing TyG index. Our study and previous studies suggest that the TyG index can be used as a simple, noninvasive prognostic indicator to help individualized stratified management of cancer patients, including HCC [19].

Our study found that the TyG index, but not other metabolism or IR-related factors such as BMI, history of diabetes, TG/HDL-c index, and the TyG-BMI index, independently predicted better OS in HCC. The results were similar to the findings of a recent research in gastric cancer [7]. However, these results warrant cautious interpretation. IR is a well-accepted risk factor in various cancers [20–22], but recent studies indicated some different evidence. For example, higher BMI was often linked to IR. However, higher BMI was found to be associated with better prognosis in lung, renal, colorectal, and liver cancers, which is the so-called “obesity paradox” [23–25]. Mechanically, reprogramming of the immune microenvironment and glucose-lipid metabolism, intra-tumoral microbiota, cancer-related cachexia, adaptation to IR, or anti-diabetes medication’s drug effect might play an important role [25–28]. The seemingly contradictory protective role of TyG in OS in certain tumors, including HCC, suggests an urgent need for more in-depth studies on the relationship and mechanisms between IR and its associated predictors in cancer.

The prognostic value of Tyg was not significantly different in most subgroups, except the ALT and major resection subgroup. Our study suggested that the predicted value of the TyG index may be attenuated in patients with higher ALT levels and patients who underwent more extensive surgical interventions. High ALT levels were linked to IR in various studies [29]. The underlying mechanisms are unclear. However, one possible explanation might be the complex interplay between liver function, hepatectomy, insulin resistance, and cancer progression [30]. More severe liver function abnormalities and major hepatectomy might alter the underlying metabolic and inflammatory profiles, inverse the prognostic value of TyG in HCC [31]. Furthermore, subgroup analysis revealed that the proportional effect of the adverse prognostic role of a lower TyG index on mortality was consistent across different subgroups. However, when focusing on the stage I + II subgroup, although no independent prognostic factors were identified, it was found that the AUC of TyG, TG/HDL-c, and the TyG-BMI index for prognostic prediction improved compared to the overall population. These results need to be interpreted with caution. The findings suggest that including the stage III + IV population might have led to an underestimation of the prognostic efficacy of these parameters. However, considering the insufficient long-term follow-up time for the studied population, the relatively high proportion of censored data, and the significant differences in case numbers across different stages, especially the limited number of stage III + IV cases, further validation is needed to determine whether there are significant differences in the prognostic role of TyG across different stage subgroups. Together, findings from our subgroup analysis highlighted the need to elucidate further the specific prognostic value of the TyG index in HCC with different subgroups.

Our study was the first to investigate the prognostic value of the TyG, TG/HDL-c, and the TyG-BMI index in patients with hepatocellular carcinoma (HCC) undergoing surgical treatment. We found that the TyG index could be an independent protective prognostic factor for HCC. Nomogram combing TyG index demonstrated good calibration, discriminative ability, and clinical benefit for HCC prognosis management. Nevertheless, several limitations do exist. First, the study's single center and retrospective design might lead to inevitable biases, like selection bias. Second, the constructed nomogram lacks external validation. Third, many patients in our study did not reach the endpoint of death, and most patients were stage I + II patients. For example, the 5-year survival rate of HCC in our cohort was over 70%, significantly higher than that reported in many previous studies. Extending our research findings to other populations requires great cautions. There is an urgent need for multi-center, prospective studies with adequate follow-up and large sample sizes to further clarify the long-term prognostic significance of the TyG index in HCC and its various subgroups.

## Conclusions

Our study found that the TyG index and TNM stage independently predict the postoperative OS of HCC. TyG index could act as an independent protective prognostic factor for HCC. Combining the TyG index with the TNM stage helped better predict postoperative OS of HCC, especially with short-term OS. However, further validation of the prognostic significance of the TyG index in different centers, larger samples, and specific subgroups is necessary.

## Supplementary Information

Supplementary file 1. Figure 1: Forrest plot of the univariate and multivariate Cox regression analysis in stage I+II HCC

Supplementary file 2. Table 1: Baseline characteristics of included TNM stage I+II patients

## Data Availability

The data that support the findings of this study are available from the corresponding author upon reasonable request.
