# Supplementary figures and images for: Triglyceride-glucose index predicts postoperative overall survival in hepatocellular carcinoma: a retrospective cohort study
Source: Discov Oncol. 2024 Nov 13;15:651. doi: 10.1007/s12672-024-01541-9 (PMC11561194; doi:10.1007/s12672-024-01541-9)

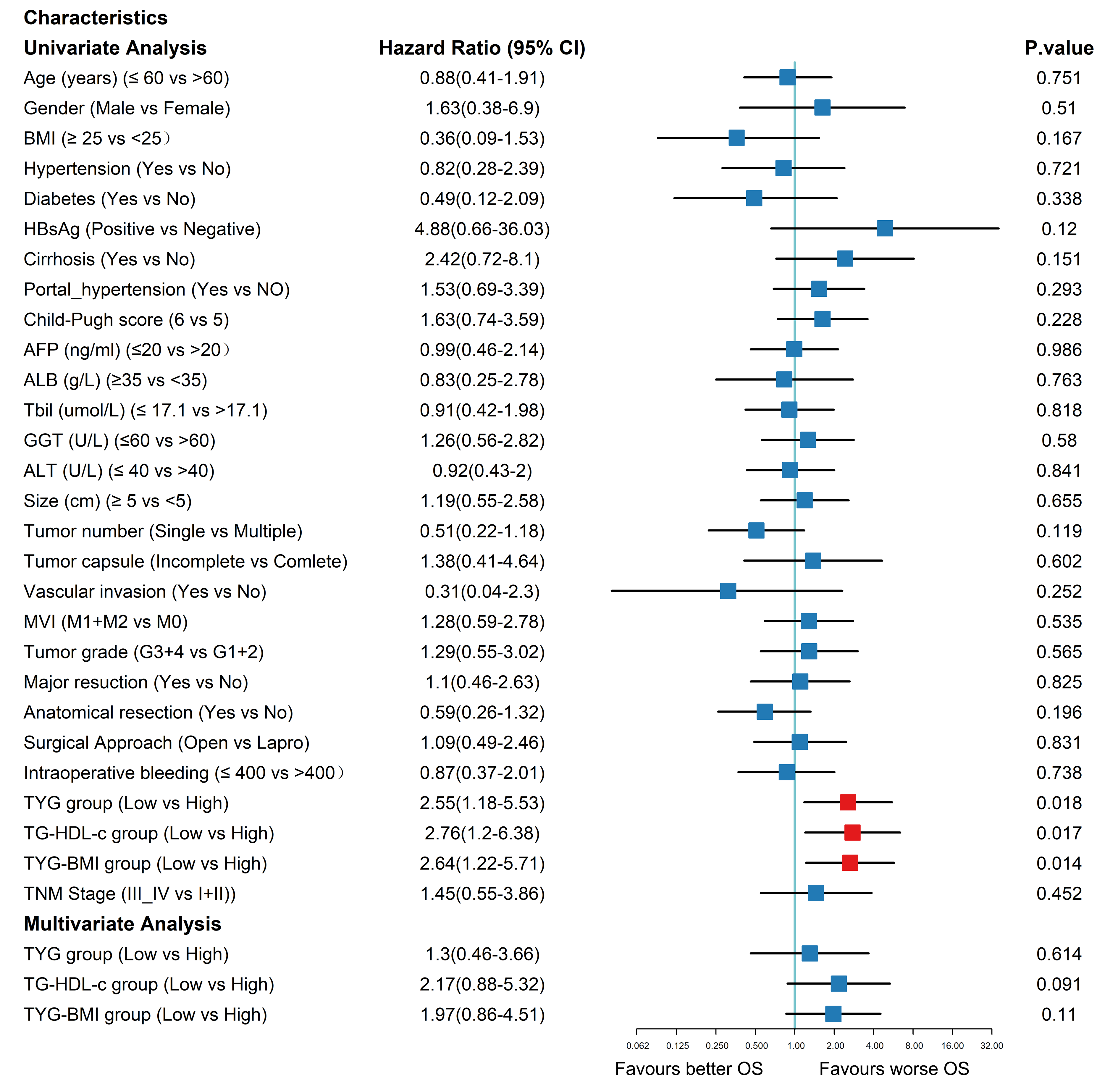

Supplement: Supplementary file 1 — Figure 1: Forrest plot of the univariate and multivariate Cox regression analysis in stage I+II HCC [file 12672_2024_1541_MOESM1_ESM.tiff]
