# Supplementary material for: Triglyceride-glucose index predicts postoperative overall survival in hepatocellular carcinoma: a retrospective cohort study
Source: Discov Oncol. 2024 Nov 13;15:651. doi: 10.1007/s12672-024-01541-9 (PMC11561194; doi:10.1007/s12672-024-01541-9)
Supplement: Supplementary file 2 — Table 1: Baseline characteristics of included TNM stage I+II patients [file 12672_2024_1541_MOESM2_ESM.docx]

| **Supplementary Table 1 Baseline characteristics of included TNM stage I+II patients** | | | | |
| --- | --- | --- | --- | --- |
| Characteristics | Overall | TyG^high^ | TyG^low^ | P |
|  | 313 | 205 | 108 |  |
| Age (mean (SD)) (Years) | 59.07 (10.44) | 61.00 [53.00, 66.00] | 59.00 [51.00, 65.25] | 0.211 |
| Gender (%) |  |  |  |  |
| Female | 34 ( 10.9) | 21 ( 10.2) | 13 ( 12.0) | 0.769 |
| Male | 279 ( 89.1) | 184 ( 89.8) | 95 ( 88.0) | |
| BMI (median [IQR]) (Kg/m2) | 22.89 (3.24) | 23.34 [21.30, 24.91] | 21.63 [19.46, 23.98] | ***<0.001*** |
| Hypertension (%) | |  |  |  |
| No | 248 ( 79.2) | 154 ( 75.1) | 94 ( 87.0) | ***0.020*** |
| Yes | 65 ( 20.8) | 51 ( 24.9) | 14 ( 13.0) | |
| Diabetes (%) |  |  |  |  |
| No | 260 ( 83.1) | 155 ( 75.6) | 105 ( 97.2) | ***<0.001*** |
| Yes | 53 ( 16.9) | 50 ( 24.4) | 3 ( 2.8) | |
| HBsAg (%) |  |  |  |  |
| Negative | 56 ( 17.9) | 46 ( 22.4) | 10 ( 9.3) | ***0.006*** |
| Positive | 257 ( 82.1) | 159 ( 77.6) | 98 ( 90.7) | |
| Cirrhosis (%) |  |  |  |  |
| No | 92 ( 29.4) | 64 ( 31.2) | 28 ( 25.9) | 0.397 |
| Yes | 221 ( 70.6) | 141 ( 68.8) | 80 ( 74.1) | |
| Portal hypertension (%) | |  |  |  |
| No | 238 ( 76.0) | 157 ( 76.6) | 81 ( 75.0) | 0.863 |
| Yes | 75 ( 24.0) | 48 ( 23.4) | 27 ( 25.0) | |
| Child-Pugh score (%) | |  |  |  |
| 5 | 222 ( 70.9) | 149 ( 72.7) | 73 ( 67.6) | 0.417 |
| 6 | 91 ( 29.1) | 56 ( 27.3) | 35 ( 32.4) | |
| AFP (median [IQR]) (ng/ml) | 1202.64 (3663.46) | 11.56 [3.89, 175.94] | 25.80 [4.69, 707.99] | 0.064 |
| ALB (mean (SD)) (g/L) | 40.93 (5.25) | 42.10 [37.70, 45.30] | 40.50 [37.98, 43.58] | 0.051 |
| Tbil (mean (SD)) (umol/L) | 18.24 (13.49) | 14.80 [10.80, 22.50] | 15.10 [12.00, 20.60] | 0.897 |
| GGT (mean (SD)) (U/L) | 94.26 (147.19) | 54.00 [33.00, 115.00] | 41.00 [25.00, 73.25] | ***0.002*** |
| ALT (mean (SD)) (U/L) | 51.97 (67.76) | 36.00 [26.00, 52.00] | 36.00 [25.00, 49.00] | 0.907 |
| FPG (mean (SD)) (mmol/L) | 5.89 (2.25) | 5.65 [5.02, 6.92] | 4.60 [4.26, 5.16] | ***<0.001*** |
| TG (mean (SD)) (mmol/L) | 1.21 (0.70) | 1.23 [1.01, 1.69] | 0.72 [0.58, 0.84] | ***<0.001*** |
| HDL-C (mean (SD)) (mmol/L) | 1.48 (0.67) | 1.25 [1.05, 1.58] | 1.44 [1.22, 1.75] | ***0.002*** |
| TG/HDL-c (mean (SD)) | 0.96 (0.71) | 0.95 [0.74, 1.35] | 0.50 [0.35, 0.63] | ***<0.001*** |
| TyG-BMI (mean (SD)) | 157.67 (28.83) | 164.61 [150.57, 184.46] | 135.40 [121.83, 152.08] | ***<0.001*** |
| Tumor size (mean (SD)) (cm) | 4.80 (2.91) | 4.00 [2.70, 6.00] | 4.30 [3.00, 6.93] | 0.097 |
| Tumor size (cm) (%) | |  |  |  |
| <5 | 186 ( 59.4) | 127 ( 62.0) | 59 ( 54.6) | 0.257 |
| ≥5 | 127 ( 40.6) | 78 ( 38.0) | 49 ( 45.4) | |
| Tumor number (%) | |  |  |  |
| Single | 256 ( 81.8) | 175 ( 85.4) | 81 ( 75.0) | ***0.035*** |
| Multiple | 57 ( 18.2) | 30 ( 14.6) | 27 ( 25.0) | |
| Tumor Capsule (%) | |  |  |  |
| Complete | 295 ( 94.2) | 193 ( 94.1) | 102 ( 94.4) | 1 |
| Incomplete | 18 ( 5.8) | 12 ( 5.9) | 6 ( 5.6) | |
| Vascular invasion (%) | |  |  |  |
| No | 278 ( 88.8) | 183 ( 89.3) | 95 ( 88.0) | 0.873 |
| Yes | 35 ( 11.2) | 22 ( 10.7) | 13 ( 12.0) | |
| MVI (%) |  |  |  |  |
| M0 | 205 ( 65.5) | 132 ( 64.4) | 73 ( 67.6) | 0.846 |
| M1 | 67 ( 21.4) | 45 ( 22.0) | 22 ( 20.4) | |
| M2 | 41 ( 13.1) | 28 ( 13.7) | 13 ( 12.0) | |
| Tumor Grade (%) | |  |  |  |
| 1 | 19 ( 6.5) | 12 ( 6.3) | 7 ( 6.7) | 0.626 |
| 2 | 208 ( 70.7) | 136 ( 72.0) | 72 ( 68.6) | |
| 3 | 65 ( 22.1) | 39 ( 20.6) | 26 ( 24.8) | |
| 4 | 2 ( 0.7) | 2 ( 1.1) | 0 ( 0.0) | |
| Anatomical resection (%) | |  |  |  |
| No | 150 ( 47.9) | 103 ( 50.2) | 47 ( 43.5) | 0.311 |
| Yes | 163 ( 52.1) | 102 ( 49.8) | 61 ( 56.5) | |
| Surgical approach (%) | |  |  |  |
| Conversion | 31 ( 9.9) | 18 ( 8.8) | 13 ( 12.0) | 0.509 |
| Laparoscopic | 215 ( 68.7) | 145 ( 70.7) | 70 ( 64.8) | |
| Open | 67 ( 21.4) | 42 ( 20.5) | 25 ( 23.1) | |
| Major resection (%) | |  |  |  |
| No | 243 ( 77.6) | 160 ( 78.0) | 83 ( 76.9) | 0.921 |
| Yes | 70 ( 22.4) | 45 ( 22.0) | 25 ( 23.1) | |
| Intraoperative_bleeding (mean (SD)) (ml) | 301.15 (383.39) | 200.00 [50.00, 400.00] | 200.00 [95.00, 400.00] | 0.642 |
| TNM stage (%) |  |  |  |  |
| I+II | 265 ( 84.7) | 176 ( 85.9) | 89 ( 82.4) | 0.523 |
| III+IV | 48 ( 15.3) | 29 ( 14.1) | 19 ( 17.6) | |

Abbreviations: ALB, albumin; ALT, alanine aminotransferase; AFP: alpha-fetoprotein; BMI: body mass index; FPG, fasting plasma glucose; HDL-c, high-density lipoprotein cholesterol; TBIL, total bilirubin; TC, total cholesterol; TG, triglycerides; TG/HDL-c: triglycerides / high-density lipoprotein cholesterol ratio; TyG, triglyceride-glucose index; TyG-BMI, triglyceride-glucose index-body mass index; GGT, glutamyl transpeptidase; HBsAg: hepatitis B surface antigen; MVI: microvascular invasion; TNM: tumor node metastasis classification.

Bold P: P<0.05.
